# Supplementary material for: Intracellular glutamine fluctuates with nitrogen availability and regulates Mycobacterium smegmatis biofilm formation
Source: J Bacteriol. 2025 Oct 8;207(11):e00252-25. doi: 10.1128/jb.00252-25 (PMC12632254; doi:10.1128/jb.00252-25)
Supplement: Supplemental figures — Figures S1 to S7. [file jb.00252-25-s0001.docx]

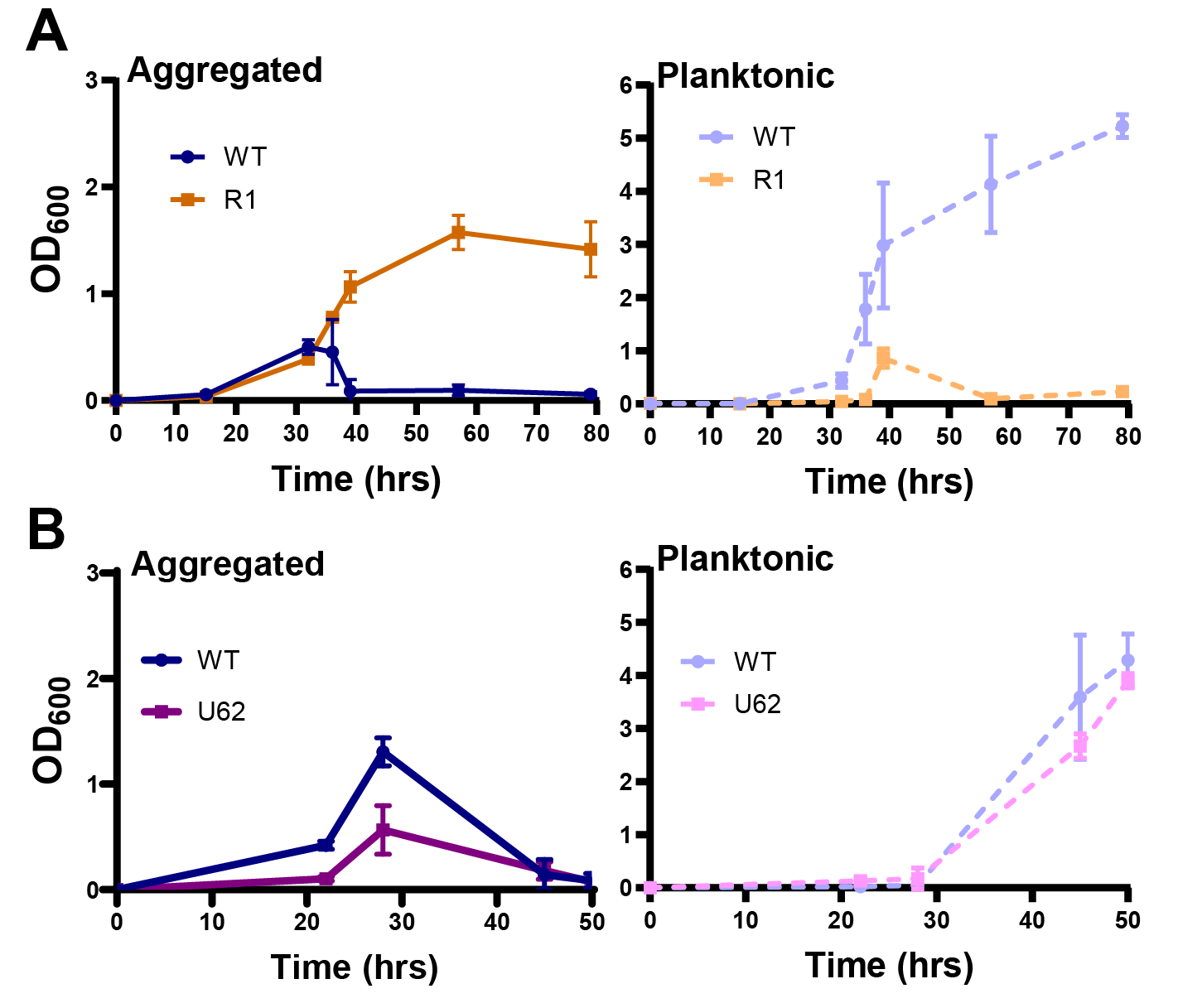


**Figure S1**– Aggregation dynamics of WT *M. smegmatis* compared to **(A)** hyper-wrinkling transductant R1 and **(B)** smooth transductant U62 in TYEM liquid medium. Error bars indicate mean $\pm$ SD (n=3).


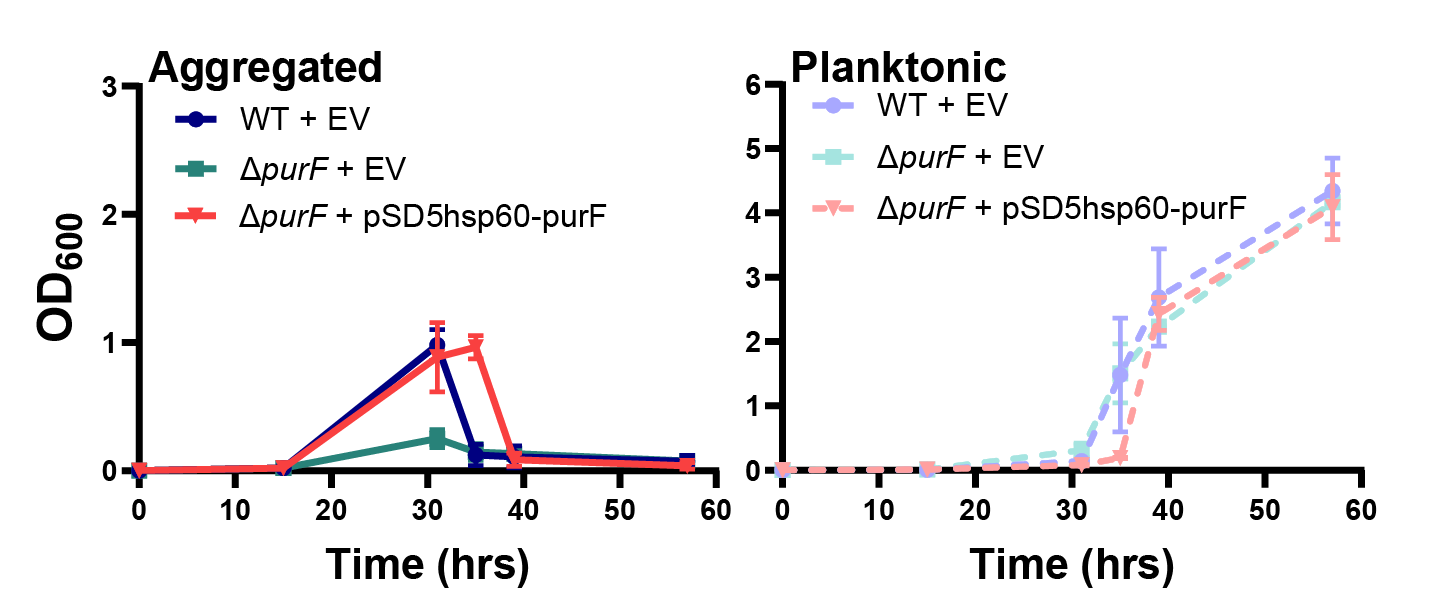


**Figure S2** – Aggregation dynamics of WT *M. smegmatis* with an empty vector (EV), Δ*purF* EV, and Δ*purF* with pSD5hsp60-*purF* complementation in TYEM liquid medium. Error bars indicate mean $\pm$ SD (n=3).


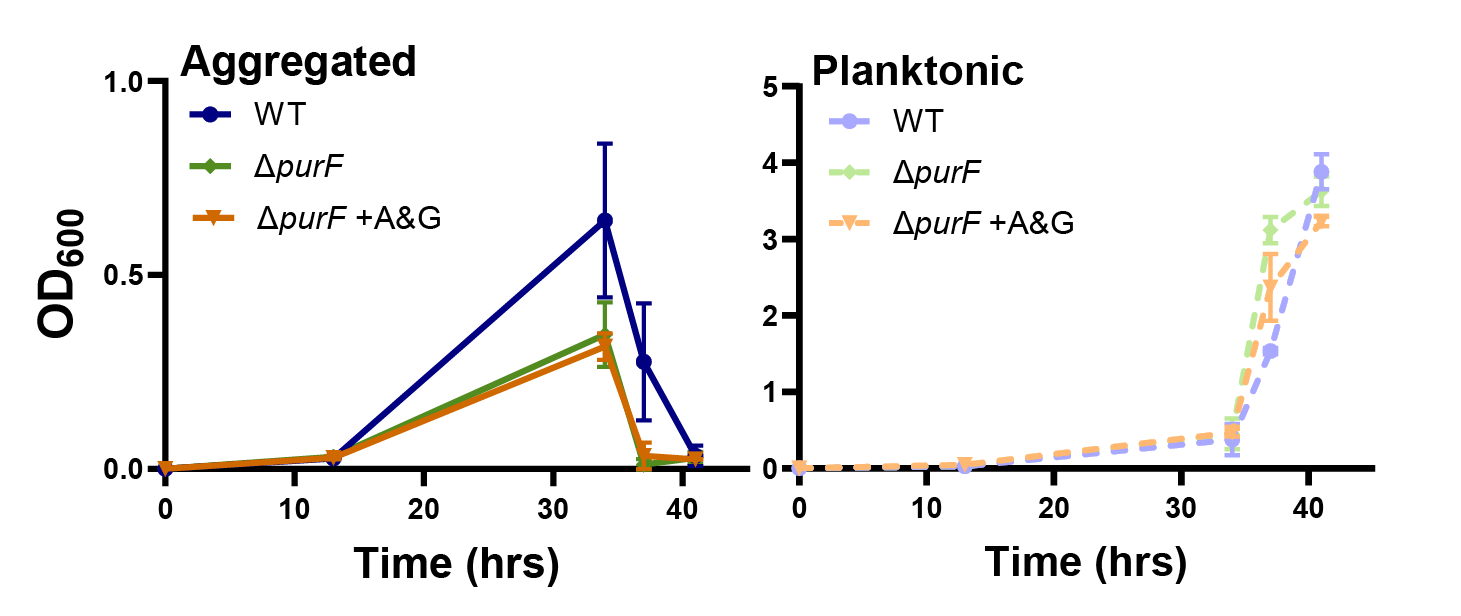


**Figure S3** – Aggregation dynamics in TYEM of WT *M. smegmatis*, Δ*purF*, and Δ*purF* with adenosine and guanosine supplementation. Error bars indicate mean $\pm$ SD (n=3).


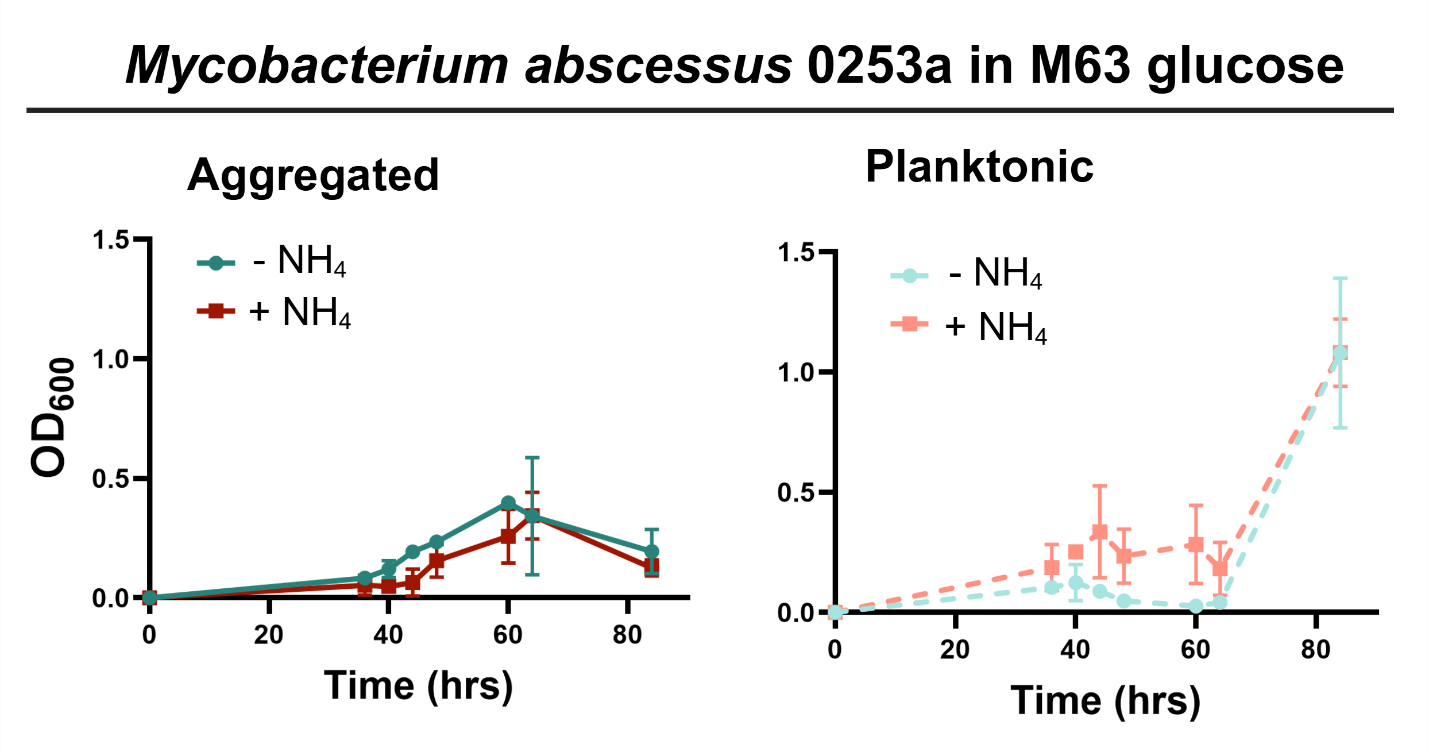
**Figure S4** – Aggregation dynamics of WT *M. abscessus* 0253a in M63 glucose medium with 5 mg/mL BSA with and without 20 mM NH_4_Cl. Error bars indicate mean $\pm$ SD (n=3).


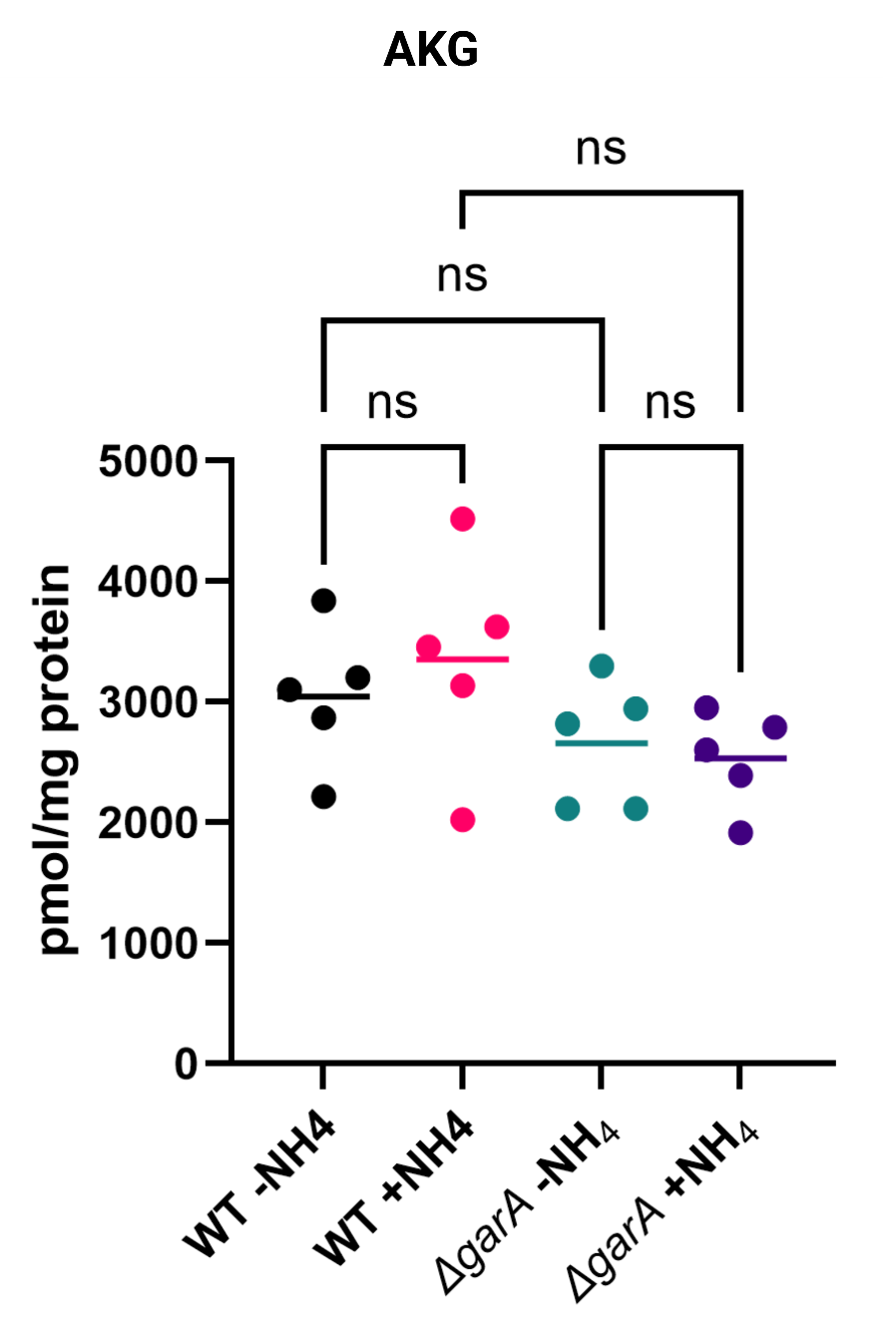


**Figure S5** – Quantification of intracellular alpha-ketoglutarate (AKG) in WT *M. smegmatis* and Δ*garA* at 40hrs of growth in M63 glucose medium with and without 20 mM NH_4_Cl. Lines indicate sample mean (n=5).


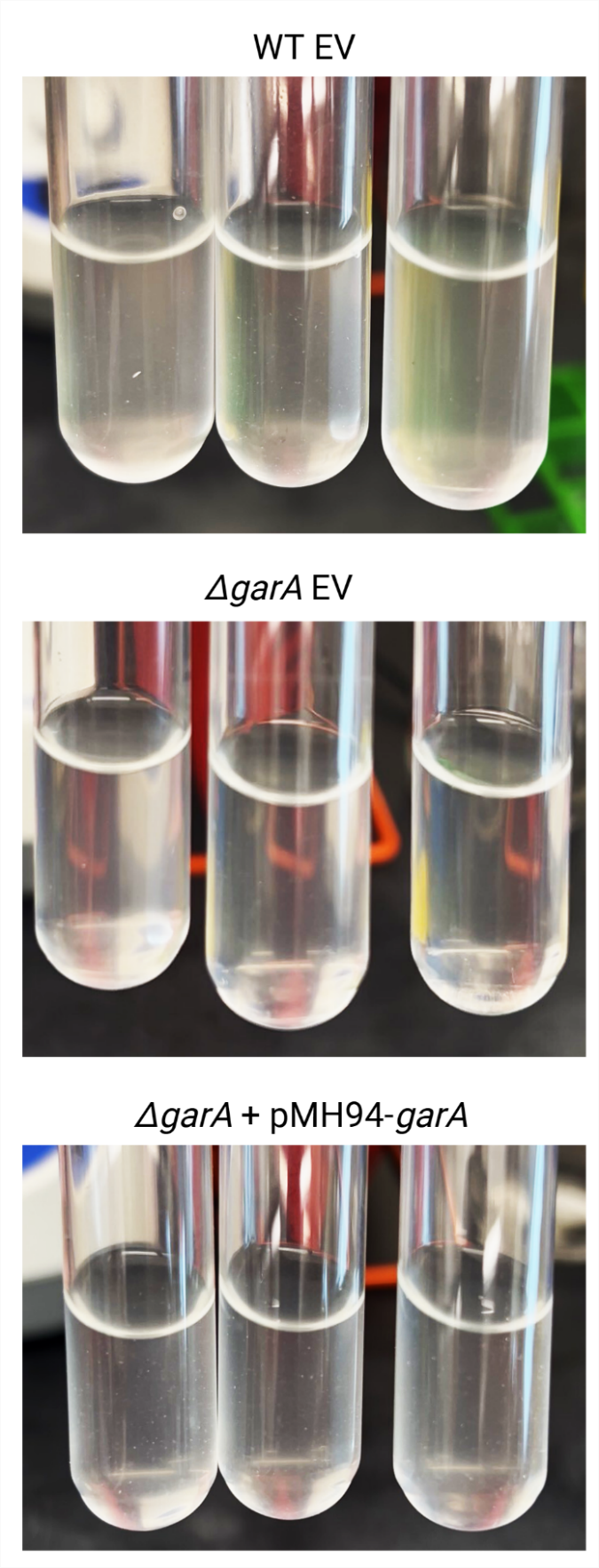


**Figure S6** – Pictures of triplicate cultures of WT + EV (pMH94), Δ*garA* + EV, and Δ*garA* + pMH94-*garA* after 24hrs of growth in M63 glucose medium without ammonium. Aggregates were observed in early stages of growth in WT and the complemented Δ*garA* strain.


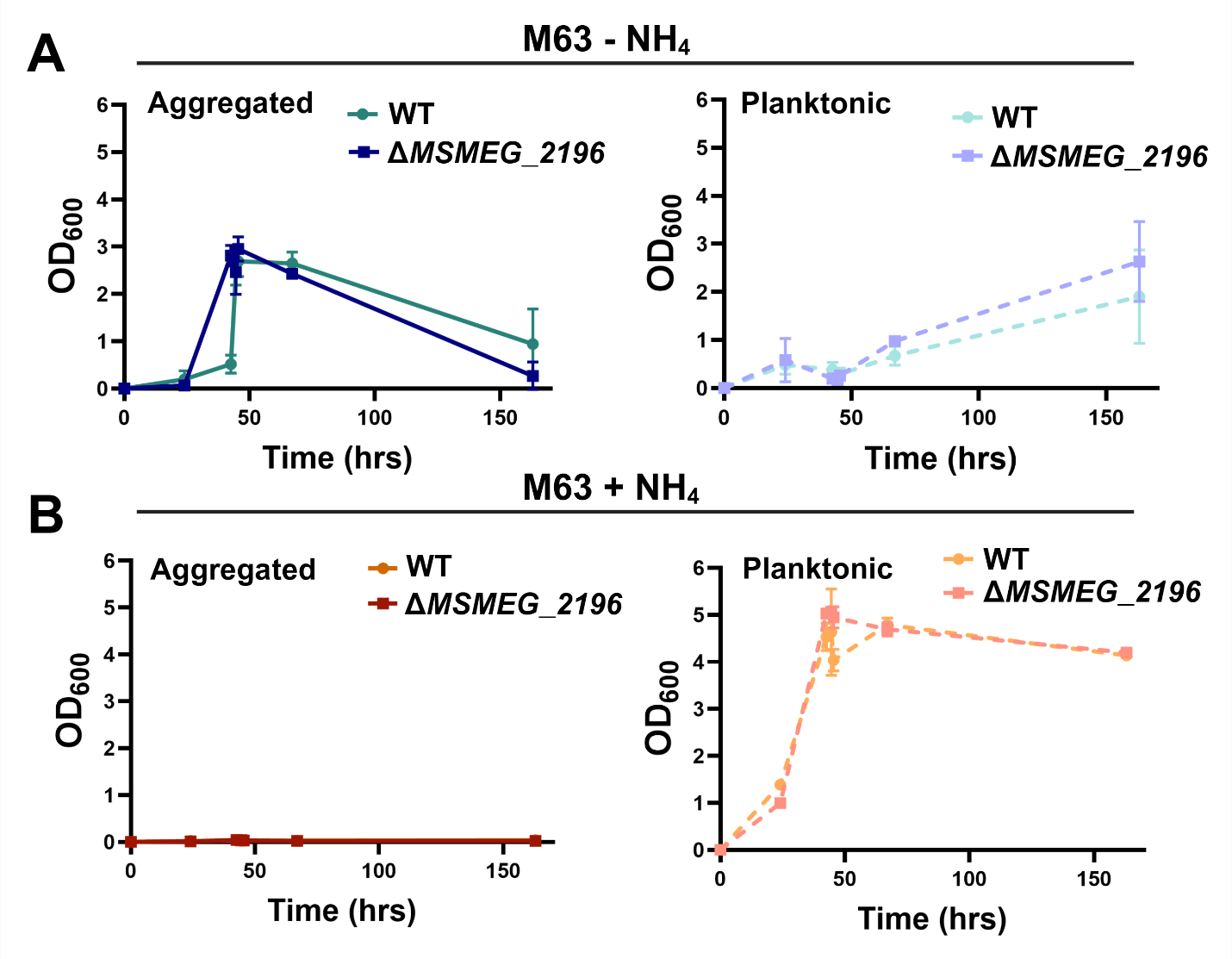


**Figure S7** – Aggregation dynamics of WT *M. smegmatis* and *ΔMSMEG_2196* in M63 glucose medium without **(A)** and with **(B)** 20 mM NH_4_Cl.
